# Risk factors for celiac disease

**DOI:** 10.1186/s13052-015-0166-y

**Published:** 2015-08-14

**Authors:** Marco Sarno, Valentina Discepolo, Riccardo Troncone, Renata Auricchio

**Affiliations:** Department of Translational Medical Science, Section of Pediatrics and European Laboratory for the Investigation of Food-Induced Diseases (ELFID), University of Naples Federico II, via S Pansini 5, 80131 Naples, Italy

## Abstract

Celiac Disease (CD) is an immune-mediated systemic disorder elicited by gluten and related prolamines in genetically susceptible individuals and it is the result of the interaction between genetic and environmental factors. Among genetic risk factors, the strongest association is with the HLA class II DQ region; nevertheless at least 39 non-HLA loci are associated with CD. Gluten is the main environmental trigger of the disease. In addition, infant feeding and weaning practices as well as timing of gluten introduction in the diet have been suggested to contribute to CD risk. Furthermore a role for infectious agents and microbiota composition in disease development has also been proposed.

Aim of this short review is to discuss the current knowledge on both genetic and environmental risk factors for the development of CD; moreover we will provide a brief overview of the possible strategies that could be envisaged to prevent this condition, at least in the population at-risk.

## Introduction

Celiac Disease (CD) is an immune-mediated systemic disorder elicited by gluten and related prolamines in genetically susceptible individuals, carrying the human leukocyte antigen (HLA)-DQ2 or HLA-DQ8 haplotypes, and characterized by the presence of a variable combination of gluten-dependent clinical manifestations, CD-specific antibodies, and a small intestinal enteropathy [1]. The pathogenesis of CD results from the interplay between genetic factors (HLA and non-HLA genes), gluten and other, still unknown, environmental triggers. Gluten peptides, modified by tissue transglutaminase, are able to elicit both an innate and an adaptive HLA-restricted gluten specific immune response in the intestinal mucosa of genetically predisposed subjects, resulting in the infiltration of the epithelium with lymphocytes and tissue remodeling leading to villous atrophy [2].

Aim of this short review is to discuss the current knowledge on genetic and environmental risk factors involved in CD pathogenesis, and the possible strategies that could be envisaged to prevent disease development, at least in at-risk subjects.

### Genetic factors

Among genetic factors associated with CD, the strongest association is with the HLA class II region. More than 90 % of patients carry one or two copies of the HLA-DQ2.5 encoded by the DQA1∗05 (alpha chain) and the DQB1∗02 (beta chain) genes. HLA-DQ2.2 is highly homologous to DQ2.5, but is associated with a lower risk because of decreased stability of bound peptides [3]. The data available on DQ2-negative CD patients indicate that they almost invariably are HLA-DQ8 positive (DQA1∗0301/DQB1∗0302). A gene dosage effect has been reported for HLA alleles, allowing classification of cases in classes of risk, the highest risk being represented by DQB1∗02 homozygosity [4]. Also recent studies conducted on big populations of infants born in high-risk families have validated the classification of cases in classes of risk [5, 6]. In particular, the Prevent CD Study Group analyzed more than 900 children from at risk families and found that CD occurred more frequently and had an earlier onset in children who were homozygous for HLA-DQ2 (DR3–DQ2/DR3–DQ2 or DR3–DQ2/DR7–DQ2) than in the other HLA risk groups [5].

A molecular hypothesis for such a phenomenon has been proposed based on the impact of the number and quality of the HLA-DQ2 molecules on gluten peptide presentation to T cells [7]. The most likely mechanism to explain the association with HLA class II genes is, in fact, that the DQ molecule binds a peptide fragment of an antigen involved in the pathogenesis of CD to present it to T cells.

The major histocompatibility complex class I region is also associated with CD risk. As a matter of fact, recently, a fine mapping of the MHC association signal has been performed and five new associations have been identified as risk factors independent of the HLA-DQ accounting for an additional 18 % of CD heritability, and providing evidence that the MHC class I region is an important locus in CD risk [8]. Of note, TcRαβ intraepithelial lymphocytes, responsible of the villous atrophy observed in CD patients’ small intestine, are restricted to MHC class I recognition. Thus the results of this study provide basis for a correlation between the MHC class I locus and the role of IELs in CD pathogenesis.

According to family studies, 87 % of total CD heritability could be explained [9], however the MHC-HLA region accounts only for 41 % of it, while the remaining is shared by an unknown number of non-HLA genes whose contribution taken singularly is modest, and estimated to be about 6 % globally. This suggests that there is still a 40 % of “missing heritability” that has not been revealed yet, but could probably be explained by common variants with very low effects or rare variants with mild effect that are not easily recognized by classical genome wide association studies (GWAS) due to lack of statistical power [10].

The identification of non-HLA risk factors can contribute to improve the identification of high-risk individuals eligible for possible future prevention strategies. In 2011, using the Immunochip platform to search for genetic association within regions of immunological interest, Trynka and colleagues [11] analyzed more than 12 thousands CD patients, not only confirming the loci that have been previously discovered by GWAS [12, 13], but most importantly identifying new susceptibility genetic variants, thus bringing the number of CD-associated risk loci to 40 including the HLA class II region. These loci are represented by 57 independent single nucleotide polymorphisms (SNPs), only 29 of which are located to coding sequences of a single gene, involved in most cases in immune processes, particularly T cell activation and recruitment, but also linked to several different biological pathways: cell differentiation, inflammation, migration [11].

In fact, as for other immune-mediated disorders, most of the identified SNPs are located in non-coding regions of the genome, suggesting that genetic regulatory mechanisms may be affected. On this basis most of recent genetic work has been focused on the study of factors that could impact on regulation of the expression of nearby protein coding genes (e-QTLs), but also of non-coding RNAs (ncRNA). Of interest, e-QTLs analysis revealed that a discrete amount of SNPs contributed to tissue-specific or specific-stimuli-triggered gene expression. Moreover it has been shown that for many immune-mediated disorders, including CD, some SNPs overlap with ncRNA regions, thus potentially contributing to differential expression of micro-RNA and long ncRNA compared to healthy subjects [10]. Of further interest, 64 % of the identified loci are shared with at least one other autoimmune disease (e.g. type 1 diabetes T1D). On this basis, the same Immunochip approach has been applied also to 14 autoimmune disorders other than CD and about 60 loci have been found to be associated to most diseases, suggesting that they might include genes controlling early and/or key processes involved in the development of autoimmunity [10].

### Environmental factors and early feeding practice

Among environmental factors the relationship between the infant feeding practices in the first year of life, particularly breast feeding and the modalities of gluten introduction (time, amount) have been the focus of several studies.

In 2006, a review and meta-analysis considered six observational case–control studies [14], finding an association between duration of breastfeeding and reduced risk of developing CD. These studies compared early feeding practices of CD patients to those of non-celiac control subjects, by using questionnaires or interviews. The authors concluded for a protective effect of breastfeeding, but at the same time identified limitations of these case–control studies, such as recall bias and the possibility of misclassification of controls, or other risk factors that could be unbalanced across children, and indicated necessity of new studies. As far as the modalities of gluten introduction are concerned, data obtained by one observational study conducted on a cohort of HLA-DR3/4 positive infants or first degree relatives of patients with T1D [15] suggested that is more cautious to introduce gluten containing food between 4 and 6 months, avoiding earlier and later introduction.

In the last few years, new relevant studies have been published, including two randomized, double-blinded trials. The Prevent CD [5] study is a multicenter, double-blinded, placebo-controlled dietary-intervention trial involving 944 children HLA-DQ2 or -DQ8 positive, with at least one first-degree relative with CD. The hypothesis was that the exposure of genetically predisposed infants to small quantities of gluten at 16 to 24 weeks of age, preferably during breastfeeding, could reduce frequency of CD at the age of 3. The participants were randomly assigned to receive daily placebo or 100 mg of immunologically active gluten from 16 to 24 weeks of age. The results showed a cumulative incidence of CD at 3 years of age similar in the gluten group and placebo group (5,9 % and 4,5 % respectively). Breastfeeding did not influence CD development. CD was significantly more frequent in the group of children who were homozygous for HLA-DQ2 than in the other HLA risk groups. Thus these results do not support the hypothesis that introduction of small amounts of gluten during breastfeeding in the window between 16 and 24 weeks of age could protect genetically predisposed infants from CD development.

The Risk of CD and Age at Gluten Introduction (CELIPREV) [6] trial was a multicenter, randomized, interventional trial that explored the relationship between age of gluten introduction and risk of CD, testing the hypothesis that gluten introduction should be delayed in infants who have a familiar risk. Newborns with a first-degree relative with CD were randomly assigned to gluten introduction at 6 months (group A) or 12 months (group B) and serology was evaluated at different times. Results showed that at 2 years of age, the prevalence of CD autoimmunity and CD was higher in group A (16%vs 7 % and 12 % vs 5 % respectively), while at 5 years these differences were no longer significant for autoimmunity (21 % vs 20 %) and overt CD (16 % vs 16 %). None of other variables studied, including breastfeeding, had a significant effect in predicting the development of CD. This study confirmed that the prevalence of autoimmunity and overt CD was significantly higher in children with high-risk HLA genotypes as compared with children with standard-risk HLA genotypes. Taken together, these studies showed that neither the timing of gluten introduction nor breastfeeding had a significant impact on the risk to develop CD; however, a delayed gluten introduction was associated with a later onset of the disease. Both studies conclude that the major risk factor of early onset is the HLA-DQ2 homozygosity [5, 6].

The Environmental Determinants of Diabetes in the Young (TEDDY) study [16] is a multicenter prospective birth-cohort study. Children were screened annually from the age of 24 months for anti-tTGA levels. During a 5 years follow-up, 12 % of the enrolled children developed CD autoimmunity and 5 % developed overt CD. Introduction of gluten before 17 weeks or later than 26 weeks was not associated with an increased risk for CD autoimmunity or CD development.

The Norwegian Mother and Child Cohort Study [17] is a prospective population-based pregnancy cohort study aimed to assess the association of CD-development with prenatal and perinatal factors (fetal growth, birth weight, mode of delivery, parental smoking, maternal education) and with early feeding practices as the age of gluten introduction and breastfeeding. In this study, a delayed gluten introduction was associated with an increased risk of CD, and a positive association between breastfeeding duration >12 months and CD risk was found compared to breastfeeding <6 months. The authors invite to cautiously interpret these data, considering a protective effect of breastfeeding limited only to early onset disease, or a possible involvement of a delayed gluten introduction associated with a higher amount of gluten in this group of children.

The Generation R [18] is another prospective cohort study, involving 1679 Dutch children positive for HLA-DQ2/DQ8, with the aim to examine the relationship between time of gluten introduction, breastfeeding and development of CD autoimmunity at 6 years of age. No association was found between breastfeeding duration or timing of gluten introduction and CD autoimmunity.

Not only early feeding practices, but also the amount of gluten introduced during weaning has been hypothesized to play a role in conferring risk of CD development. During a period between 1985 and 1994 (the so called “Swedish epidemics”) the incidence of CD rose from 65 per 100,000 to 198 per 100,000. Such a rise in CD-incidence has been attributed to an increase in the amount of gluten content in infant formulas and with a decrease in breastfeeding practice. The Exploring the Iceberg of Celiacs in Sweden (ETICS) [19] study compared two birth cohorts of 12 years old children, born in 1993, during the celiac epidemic, and born in 1997 (the epidemics ended in 1995), investigating the prevalence of CD and feeding habits. The main difference was the amount of gluten in baby food (38 g/child/day vs. 24 g/child/day). Moreover, women in the 1997 cohort breastfed for a longer period of time after having introduced gluten as compared with the 1993 cohort, (9 vs. 7 months, P < 0.001), and a significantly larger proportion of women continued breastfeeding beyond gluten introduction (78 % vs. 70 %, respectively; P < 0.001). The total prevalence of CD was 29 in 1000 and 22 in 1000 for the 1993 and 1997 cohorts, respectively. The findings of the ETICS study suggest that high amount of gluten introduced during the first year of life may contribute to the risk of CD, although the small differences in the final prevalence request caution.

A practical guide including recommendation of early feeding practice and timing of gluten introduction is reported in Table 1.

**Table 1:** Early feeding practice and risk of CD

● It should be considered that data concerning early feeding practices have been obtained from at-risk families (where at least one CD patient was present), and that possible recommendations should apply only to genetically susceptible individuals. (Of note, one third of European infants is at-risk of CD).
● No evidence that any breast feeding, its duration or persistence at the time of gluten introduction, has an effect on CD risk. However breastfeeding remains recommended.
● Early introduction of small amount of gluten does not induce tolerance.
● Later gluten introduction delays CD onset, but does not prevent its occurrence.
● Weak evidence that high amount of gluten at weaning increases CD risk, but it seems reasonable to discourage consumption of high amounts of gluten in the first year of life.

### Other environmental factors: is there a role for infectious agents?

The possible role of infectious agents is suggested by several pieces of evidence, firstly CD has a seasonal pattern. Data extracted from the population based incidence register of CD in Swedish children diagnosed from 1973 to 1997 showed that the incidence of CD in children below 2 years of age was higher if they were born during the summer/spring season as compared with the winter/autumn season. This pattern was more pronounced for boys respect to girls, suggesting an interaction between environmental factors and gender. It is possible to suppose that children born in the summer time have been in uterus during the winter period, when the rate of maternal infections is higher. Furthermore they are weaned, including gluten introduction, during winter, when the infections risk is higher. The absence of correlation between seasonality and CD risk in children diagnosed after 2 years of age suggests that environmental factors could have an impact on early immune system development, especially during the first years of life [20].

The role of early infections was explored in a population-based incident-case referent study conducted in Sweden. Having three or more parental-reported infections, regardless of the type of infection, during the first six months of life was associated with a significantly increased risk of CD, and this remained after adjusting for infant feeding and socioeconomic status. As predicted, the risk to develop CD increased synergistically in infants with a history of repeated infections and large amounts of gluten introduced after discontinuing breastfeeding [21].

Specific infectious agents are considered as possible environmental factors triggering autoimmune diseases, having a role in gut permeability alteration and immunity activation. The adenovirus serotype 12 (Ad12) was associated with CD. Immunological cross reactivity of a Ad12 peptide within the alpha-gliadin was proposed as link between Ad12 infection and CD triggering [22], but further studies did not support this hypothesis. In 2006, a longitudinal study involving children carrying the CD-associated HLA risk alleles explored correlation between CD autoimmunity development and rotavirus infections, detected as rotavirus antibodies increase. Results showed that frequent rotavirus infections predicted a higher risk of CD autoimmunity [23].

Furthermore, increasing evidences support a role of the intestinal microbial environment and the development of the disease. An inverse relationship between Helicobacter Pylori and CD has been also reported. The observation that children who were born by elective caesarean section have an increased risk in comparison to those born by vaginal delivery has also been put in relation with the different composition of the intestinal microbiota. As a matter of fact, differences in microbiota composition between CD patients and healthy controls have been described. In particular, an increase in Bacteroides species (spp) and reductions of Bifidobacterium spp. were found in untreated CD patients and those differences persisted in part despite adherence to a GFD. E. Coli and Staphylococcus were also higher in untreated CD patients, but these differences disappeared upon a GFD. Intestinal microbiota composition has an important role in gut immunity modulation, thus intestinal dysbiosis could possibly impact on the risk of CD development. However it remains unclear whether changes in microbiota are a cause or a result of intestinal inflammation [24].

## Conclusions

The impact of genetic factors on the development of CD is strongly supported by the high concordance rate in monozygotic twins [9] and further emphasized by the results of the prospective studies conducted in at-risk cohorts, mainly represented by infants born in families with at least one celiac member. On the other hand, the relevance of environmental factors in triggering CD is suggested by the significant changes in the incidence of the disease by time and place. Evidence from the United States [25] and from Finland [26] suggest that the sero-prevalence of CD as defined by high levels of anti-tTGA and anti-endomysium antibodies has significantly increased in the last decades at a rate that cannot be attributed to the genetic drive. The increase is coherent with the hygiene hypothesis, which attributes to the decreased exposure to microbes and the change in microbiota the driving effect of the rise of autoimmune and atopic conditions. Furthermore, the possible role played in pathogenesis by innate cytokines, such as type one interferon, has suggested the possible involvement of viral infections in disease onset. Infectious episodes, especially gastrointestinal infections, could potentially contribute to CD pathogenesis as they might induce an increase in gut permeability with higher antigen penetration and/or may drive the immune system towards a pro-inflammatory T helper 1 response.

Regarding feeding patterns in the first year of life, recent studies have failed to show an impact of breastfeeding duration or timing of gluten introduction on disease development, whereas the data from Sweden point to a possible role of high amount of gluten ingested during the first year of life. All these elements have been integrated by Koning [7] in an attractive model according to which the amount of immunogenic gliadin peptides presented by APC, the higher expression of HLA-DQ and, possibly, conditions promoting the induction of inflammatory T cell responses, such as a viral infection, may have an impact on the risk of CD [27]. Prevention strategies could be implemented, particularly in families at-risk, based on the reduction of immunogenic gluten and, if the relevance of infections is confirmed, on programs of vaccination. In particular the identification of wheat varieties with lower pro-inflammatory activity [28] or with less immunogenic sequences [29], could lead to initiatives to favor their diffusion among the general population with the result of an overall decline in the prevalence of CD.

## Abbreviations

CD: Celiac disease
HLA: Human leukocyte antigen
GWAS: Genome wide association study
T1D: Type-1 diabetes
tTGA: Tissue transglutaminase antibodies
spp: Species
